# Neonatal Ilio-Psoas Abscess: Report of Two Cases

**Published:** 2014-01-01

**Authors:** Minakshi Sham, Dasmit Singh

**Affiliations:** Department of Paediatric Surgery, BJ Medical College; PUNE – 411 001

**Keywords:** Iliopsoas abscess (IPA), Neonate, Limb deformity, Extraperitoneal surgical drainage

## Abstract

Ilio-psoas abscess (IPA) is rare in children and exceptional in the neonate. However, we recently managed two consecutive male neonates with right-sided IPA. The first baby was born two days after rupture of the membranes and had thick meconium-stained amniotic fluid. There was no such high risk factor in the second child. Diagnosis was made by ultrasonography in both the patients. Extraperitoneal surgical drainage was done and systemic antibiotics were given. Delay in presentation and uncontrolled sepsis, led to mortality in the first case. On the contrary, relatively early presentation, prompt drainage of the abscess and good response to higher antibiotics, lead to successful salvage of the second baby.

## INTRODUCTION

IPAs can be described as either primary or secondary. Primary IPAs have no detectable source and are the most common type in children. The most common organism in Primary IPA is Staphylococcus Aureus, indicating that an unidentified, cutaneous source leading to bacteremia is most likely. Causative organisms implicated in secondary IPAs in children are Staphylococcus Aureus, Streptococcus Pneumoniae and Escherichia Coli [2]. Though IPAs are occasionally reported in younger children hailing from the developing tropical countries [3], presentation in a neonate is extremely rare [4,5]. We report two neonatal cases of right-sided IPAs managed by us. 

## CASE REPORT

**Case 1:**A full-term, male neonate weighing 2.7kg was born vaginally 2 days after rupture of the membranes. The amniotic fluid was thick-meconium stained. On day 6 of life, swelling of the right hip was noted. The child was brought to us 6-7 days thereafter. He had fever on and off of 3 days’ duration. There was history of concurrent respiratory infection and refusal to feed. BCG vaccination was given on day 9 of life. The child had not received any intramuscular injection over the affected thigh. There was no history of trauma, femoral vein catheter insertion and omphalitis. 

On examination, the right hip was swollen with abduction deformity of the right hip (Fig. 1). There was limitation of range of movements. The abdomen appeared protuberant with thinned out, shiny skin and visibly dilated veins. He had bilateral scrotal edema, more on the right side. Oral thrush was noted, though the child was exclusively breast-fed. He also had a small abscess over the dorsum of right hand (Fig. 1).

**Figure:**
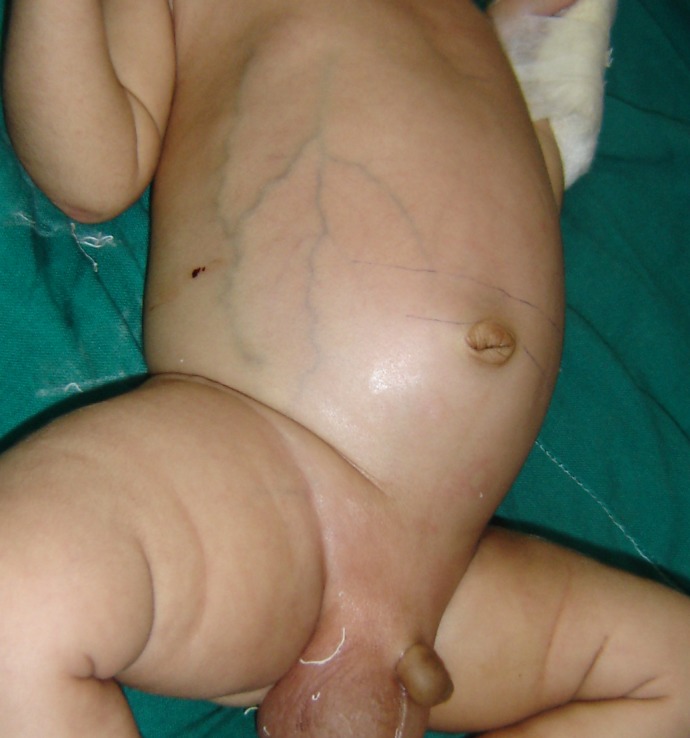
Figure 1: Leg deformity and ipsilateral scrotal swelling.

The child had hemoglobin of 13 gm%, WBC Count was 21,000/cmm and Platelet count was 65,000/cmm. There was no acidosis, however baseline PO2 was low (80). His serology tests (HIV, HBsAg) were negative. Bilateral lower zone infiltrates were noted on chest X-Ray, whereas X-Ray of the abdomen and pelvis showed no specific pathology. Ultrasonography revealed a large abscess measuring 8.6× 7.3× 4.8 cm involving the right ilio-psoas muscle and displacing the right kidney. Since ultrasound-guided percutaneous needle aspiration failed, extraperitoneal surgical drainage of the abscess was done. It drained about 50 cc of frank pus. The baby received intravenous Meropenem; but succumbed to sepsis on post-operative day 2. Pus culture had grown Staphylococcus Aureus. 

**Case 2:**A 21-day-old male neonate was brought with history of swelling over the right inguinal region (Fig. 2). There was swelling and obvious cutaneous edema over the right lumbar region as well. Limitation of leg movement and abduction deformity of the right hip was noted on examination. There was no history of trauma, femoral vein catheter insertion and omphalitis.

**Figure:**
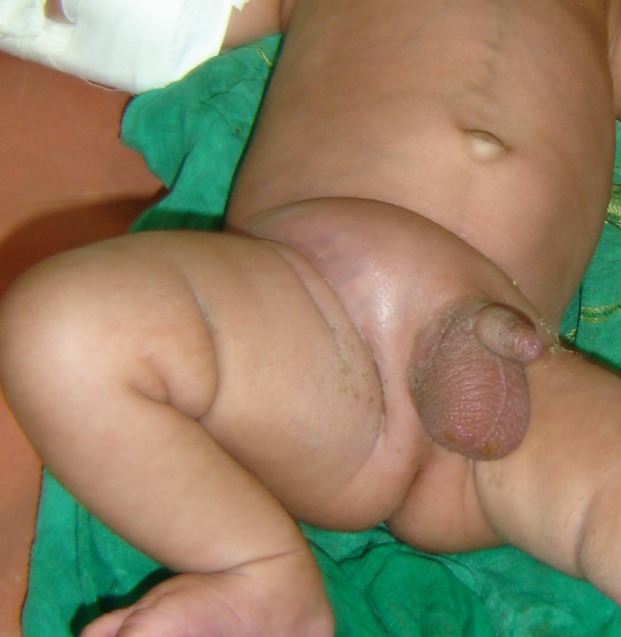
Figure 2: Swelling over the right inguinal region as the presenting feature of ilio-psoas abscess.

His hemoglobin was 16 gm%, WBC Count was 12,000/cmm and Platelet count was normal. The serology was negative. X-Ray of the spine and both hips was normal. Ultrasonography revealed abscess of the right ilio-psoas muscle. The child was put on Ampicillin and Cloxacillin. Extraperitoneal surgical drainage was done under anaesthesia. It drained about 30 cc of frank pus. A tube drain was kept in-situ. 

Post-operative course was uneventful. Breastfeeds were started on POD1 and were well tolerated. Wound healing was good except for minor superficial wound infection. Culture of pus from the abscess cavity showed Staphylococcus Aureus. Presently at a follow up of 2 years, he is thriving well and has attained age appropriate milestones. There is no limb deformity and the gait is normal.

## DISCUSSION

IPA is rare in infants and young children [4]. The major presenting symptoms are leg or groin swelling, limitation of leg motion and pain [5]. They may present as a tender mass palpable in the iliac fossa, lower abdomen, pelvis or the inguinal region. Peritoneal irritation is uncommon since the fascia covering the psoas muscle prevents spreading of the abscess to retroperitoneum or free peritoneal space. Fever, elevated ESR and leukocytosis are almost always the presenting signs. Refusal to feed may be noted especially in neonates.

High-resolution ultrasound or a Computed Tomography (CT) Scan confirms the diagnosis of IPA. In neonates, it is difficult and crucial to differentiate IPA from septic arthritis of the hip since the management of both is different. Ultrasonography can provide the correct diagnosis in such a situation in neonates. The standard management of IPA consists of intravenous antibiotics and adequate drainage - either percutaneous [6] or operative. Surgical drainage has been found to be superior in achieving prompt recovery by Santaella et al [7]. Katara and others have reported successful use of laparoscopy (retroperitoneoscoy) for drainage of these abscesses [8].

Our report describes two cases of IPA in neonates. In the first baby, presenting symptoms were thigh and scrotal swelling, limitation of leg movement, concurrent cutaneous and respiratory infection with fever and refusal to feed. Whereas, swelling over the right inguinal region was the main presenting feature in the second child. Peri-natal infection because of prolonged rupture of fetal membranes and sepsis secondary to cutaneous infection over the right hand were the possible etiologic factors in the first neonate; whereas the second child had no such obvious factors. Abscess of the right ilio-psoas muscle was diagnosed on ultrasonography in both the patients. Cultures of pus from the abscess cavity in both the patients had grown Staphylococcus Aureus. 

The insidious and occult nature of abscess had caused diagnostic delay, prolonged sepsis, and was the cause of mortality in the first case. Whereas in the second case relatively early presentation, prompt drainage of the abscess and good response to higher antibiotics, salvaged the baby. 

## CONCLUSION

Since morbidity and mortality are related to delay in management, a high index of suspicion is required for IPA if a neonate presents with limb disuse and fever of unknown origin. We urge fellow colleagues managing neonates to add IPA to the list of differential diagnoses in a baby presenting with fever with unknown focus and refusal to feed so as to institute early treatment and help prevent neonatal mortality. Similarly, measures like starting intravenous antibiotics to a mother with premature rupture of membranes, admitting babies born to these mothers for observation and in general, strict adherence to principles of asepsis during delivery and routine handling of the neonate in perinatal period would go a long way to salvage these neonates.

## Footnotes

**Source of Support:** Nil

**Conflict of Interest:** None

